# Alterations in the white matter structure of major depressive disorder patients and their link to childhood trauma

**DOI:** 10.3389/fpsyt.2024.1364786

**Published:** 2024-03-04

**Authors:** Haomian Zhao, Bei Rong, Guoqing Gao, Mingzhe Zhou, Junhua Huang, Ning Tu, Lihong Bu, Ling Xiao, Gaohua Wang

**Affiliations:** ^1^ Department of Psychiatry, Renmin Hospital of Wuhan University, Wuhan, Hubei, China; ^2^ Institute of Neuropsychiatry, Renmin Hospital of Wuhan University, Wuhan, Hubei, China; ^3^ PET-CT/MR Center, Renmin Hospital of Wuhan University, Wuhan, Hubei, China; ^4^ Taikang Center for Life and Medical Sciences, Wuhan University, Wuhan, Hubei, China

**Keywords:** major depressive disorder, childhood trauma, diffusion tensor imaging(DTI), fractional anisotropy, tract-based spatial statistics(TBSS)

## Abstract

**Objectives:**

Major Depressive Disorder (MDD) is significantly influenced by childhood trauma (CT), affecting brain anatomy and functionality. Despite the unique disease trajectory in MDD patients with CT, the underlying neurobiological mechanisms remain unclear. Our objective is to investigate CT’s impact on the white matter structure of the brain in patients with MDD.

**Methods:**

This research employed tract-based spatial statistics (TBSS) to detect variations between groups in Fractional Anisotropy (FA) throughout the whole brain in 71 medication-free MDD patients and 97 HCs. Participants filled out the Childhood Trauma Questionnaire (CTQ) and assessments for depression and anxiety symptoms. The relationship between FA and CTQ scores was explored with partial correlation analysis, adjusting for factors such as age, gender, educational background, and length of illness.

**Results:**

Compared to HCs, the MDD group showed decreased FA values in the right posterior limb of the internal capsule (PLIC), the inferior fronto-occipital fasciculus (IFOF), and bilateral superior longitudinal fasciculus (SLF). Simple effects analysis revealed that compared to HC-CT, the MDD-CT group demonstrated decreased FA values in right PLIC, IFOF, and bilateral SLF. The MDD-nCT group showed decreased FA values in right PLIC and IFOF compared to HC-nCT. The total scores and subscale scores of CTQ were negatively correlated with the FA in the right SLF.

**Conclusion:**

The right SLF may potentially be influenced by CT during the brain development of individuals with MDD. These results enhance our knowledge of the role of the SLF in the pathophysiology of MDD and the neurobiological mechanisms by which CT influences MDD.

## Introduction

Major Depressive Disorder (MDD) is a mental illness linked to severe consequences, including impaired social and occupational functioning, substance dependence, and suicide. Its core symptoms are persistent depressive mood and loss of interest (1). Globally, MDD impacts around 16% of individuals and stands as a primary contributor to disease burden (2, 3). The neurobiological mechanisms of MDD are not fully elucidated, and exploring these mechanisms and finding effective treatments are crucial.

It is well-known that the onset of MDD has many potential factors, with childhood trauma (CT) being the known risk factor (4). Increasing evidence suggests that people with a past of CT are more susceptible to psychiatric illnesses, including MDD (5), bipolar disorder, and post-traumatic stress disorder (6). Studies indicate that 46% of individuals diagnosed with MDD have experienced CT (7). Moreover, CT encompasses various forms such as emotional abuse, physical abuse, emotional neglect, and physical neglect; various types of CT may further elevate the risk of adverse outcomes later in life, like how physical abuse might affect an adult’s responsiveness to drug treatment (8). The impact of CT on MDD has been validated, and clinical research has shown that MDD patients with a background of CT exhibit higher suicide risks, increased severity of depressive symptoms, and functional deficits (9). Plentiful research indicates that CT is connected with immune, endocrine, and epigenetic activities, along with alterations in brain anatomy and functionality (10, 11). Despite the unique disease trajectory of MDD patients with CT, its neurobiological mechanisms remain unclear.

With the advancement of neuroimaging technologies, MRI studies suggest that healthy individuals with a history of CT show widespread cortical and subcortical morphological changes. Studies involving gray matter regions have found an association between CT experience and lower volumes of the hippocampus and amygdala (12–14). Beyond gray matter regions, several studies have reported specific white matter(WM) changes in individuals with an experience of CT, including the inferior longitudinal fasciculus, uncinate fasciculus, thalamic radiation, corona radiata, longitudinal fasciculi, cingulum, and corpus callosum (15–17). Notably, there is an anatomical overlap between the specific WM change areas in MDD patients and those mentioned above. This overlap might validate the close connection between CT and MDD, though it might also suggest a need to discern whether these changes are attributable to CT or MDD. Therefore, studying the impact of CT on MDD patients is necessary.

Many studies have proven that MDD patients with an experience of CT exhibit specific functional changes in the brain (18–21). However, only a few studies have explored the morphological changes in the brains of MDD patients with CT. These structural MRI study results are inconsistent (22, 23), with some finding changes and others not, possibly due to heterogeneity in study subjects (such as age, medication, duration of illness) and methodological differences in previous research. The effect of antidepressants on WM is unclear, as most past studies included medicated patients, making it difficult to determine whether these results are confounded by medication effects. Moreover, chronic or extended-duration illnesses may result in more pronounced neuroimaging alterations, and drug-naive patients with a shorter illness duration might help us better understand the early onset of the disease. Additionally, many studies merely demonstrate the impact of CT on the WM structure of the brain in MDD patients through correlation analysis (24, 25). Our article will refine the grouping based on CT and, on this basis, further investigate the effects of CT on participants and its interaction with the MDD diagnosis.

Diffusion Tensor Imaging (DTI) is among the most commonly employed techniques to assess the integrity of MDD brain WM, helping us better explore the disease’s underlying biological mechanisms and improve consensus on diagnosis and treatment (26). Fractional anisotropy (FA) is particularly responsive to alterations in microstructure and is often used to examine WM structural connectivity in MDD (27). Impaired WM integrity might be one of the most important pathogenic mechanisms in depression with CT, and it’s crucial to explore which fiber tracts are affected and whether WM structural changes mediate the relationship between CT and emotional/cognitive functional impairment. Among the primary analysis methods in DTI, region of interest (ROI) analysis and voxel-based morphometry present clear drawbacks: ROI analysis might restrict results to ‘regions of interest’, potentially overlooking other significant brain regions; voxel-based morphometry faces issues like registration, smoothing, and other MRI data processing problems (28). Tract-Based Spatial Statistics (TBSS) can comprehensively explore all brain regions associated with changes related to CT and is more sensitive, objective, and interpretable in DTI data analysis.

In this study, the TBSS method will be used to explore how CT and MDD affect WM structural changes and whether there is an interaction between them in medication-free MDD individuals versus comparable HCs. Additionally, we examined whether CT affects mood-related aspects by mediating changes in WM. Participants’ levels of depression and anxiety were assessed using depression and anxiety scales. Based on this, we further investigated the correlation between WM structure and the severity of depression, anxiety symptoms, and the extent of CT. We hypothesize that CT may have long-term and sustained impacts, associated with structural changes in the brains of adult patients with MDD.

## Materials and methods

### Participants

This research encompassed 71 drug-naive MDD patients and 97 healthy controls (HCs), matched for gender, age, and educational level. MDD patients were sourced from the outpatient psychiatry department of Renmin Hospital of Wuhan University between April 2021 and September 2023. HCs with no history of psychiatric disorders were recruited from the community through advertisements. Diagnoses of MDD were made by psychiatrists through the Structured Clinical Interview for DSM-V (SCID). Entry requirements for the MDD group included: (1) fulfilling DSM-V standards for a major depressive episode; (2) first-episode, treatment-naive for psychotropic drugs or psychotherapy; (3) aged between 18-55 years; (4) Han Chinese ethnic;(5) right-handed; (6) ≥9 years of education. HCs fulfilled these conditions: (1) absence of significant psychiatric conditions or a family history of major mental disorders; (2) aged 18-55 years; (3) right-handed. Exclusion criteria were: (1) a record of other DSM-V Axis I psychiatric issues; (2) past or present organic brain disease, brain trauma, or cranial surgery; (3) history of substance abuse or dependence; (4) contraindications for MRI scanning; (5) pregnant or lactating women. On the day of scanning, all participants’ depression and anxiety levels were evaluated using the 24-item Hamilton Depression Rating Scale (HAMD-24) and the Hamilton Anxiety Rating Scale (HAMA).

Demographic and clinical characteristics of all participants were collected. The Childhood Trauma Questionnaire (CTQ) in its Chinese variant was utilized to evaluate participants’ childhood experiences (29). This questionnaire comprises 28 items assessing CT across five subscales: Emotional Neglect (EN), Physical Neglect (PN), Sexual Abuse (SA), Emotional Abuse (EA), and Physical Abuse (PA). Thresholds for moderate to severe trauma on each subscale were: PN≥10, PA≥10, SA≥8, EN≥15, EA≥13. In our research, participants with scores exceeding any of the thresholds were considered to have been exposed to moderate or severe CT, while those scoring below were considered to have none or mild CT. Participants were divided into four groups: MDD patients with moderate or severe CT (MDD-CT), MDD patients with none or mild CT(MDD-nCT), HC with moderate/severe CT(HC-CT), and HC with none or mild CT(HC-nCT). All recruited participants gave informed consent for this clinical research, which received approval from the Ethics Committee of Renmin Hospital of Wuhan University.

### MRI acquisition

MRI data for all participants were collected using a 3.0T GE Signa HDx MRI scanner at Renmin Hospital of Wuhan University. Participants were instructed to lie quietly in a supine position on the scanner, with head movements minimized by padding. T1-weighted structural images were captured using the subsequent parameters: repetition time; 8.5 ms; flip angle, 12^°^ echo time, 3.2 ms; slice thickness, 1.0 mm; gap, 0.0 mm; field of view, 256 mm × 256 mm; matrix, 256 × 256; voxel size, 1.0 mm × 1.0 mm × 1.0 mm; 176 slices. Diffusion tensor images were acquired by employing spin echoplanar imaging (EPI) sequences with these parameters: repetition time, 2000 ms; flip angle, 90^°^; echo time, 30 ms; voxel size, 3.4 mm × 3.4 mm × 4.0 mm; slice thickness, 4.0 mm; slice gap, 0 mm; FOV = 220 mm × 220 mm; matrix size, 64 × 64; 36 slices.

### MRI data preprocessing

TBSS is a method for exploratory analysis that relies on image registration and analysis of the entire brain at the voxel level. All DTI images were processed using functional MR imaging of the brain (FMRIB) Software Library (FSL) (http://www.fmrib.ox.au.uk/fsl).

The preprocessing procedure for TBSS in neuroimaging involves several key steps to prepare diffusion data for analysis. Initially, high-quality DTI data is acquired, which is then corrected for motion and eddy current distortions. Following this, brain extraction tools, like FSL’s BET, are used to remove non-brain structures from the DTI images. Subsequently, diffusion measures such as FA are computed.

After generating FA images for each subject, they were aligned to the FMRIB58_FA template and transformed into the MNI standard space through affine registration. This process produced standard space versions of individual FA images, which were then averaged to form a mean FA image. A threshold of 0.2 for FA was set to omit voxels with low FA values. This resulted in a mean FA skeleton representing the center of all WM tracts. Finally, all individual standard space FA images were mapped onto the mean FA skeleton. Then we create a 4D file composed of all individual FA skeletons. Fslmaths, a utility command line from FSL, was frequently utilized to delineate exact regions of interest where notable differences between patient and HCs were present.

### Statistical analysis

Demographic data of MDD patients and HCs, including age, gender, age, gender, education, illness duration and family history, depression level, anxiety level, CTQ scores, and imaging data, were compared. Categorical data were presented as proportions, and continuous variables as means and standard deviations. Shapiro-Wilkes test for normality and Levene’s test were used to assess the distribution and homogeneity of variances using SPSS 27.0. Gender family history differences were assessed using Chi-square tests. Demographic and behavioral differences in the two groups were assessed via independent sample t-tests or Mann-Whitney U tests (when the distributions were not Gaussian). One-way ANOVA tests with Bonferroni-test or Welch-ANOVA with Games-Howell post-tests (for unequal variances) were employed to compare these variables among the four groups.

For imaging data, two-way between-subjects ANOVA was employed to analyze data from the four groups, using FSL Randomize tool with 5000 permutations with the threshold-free cluster enhancement option (TFCE). Using this to examine the effects of diagnosis and CT, and to determine if there is an interaction between diagnosis and CT.

To examine whether clinical characteristics of MDD patients were related to their WM integrity, partial correlation analysis was conducted while adjusting for age, gender, education, and illness duration. The correlation between the average FA values and the patients’ levels of depression, anxiety, total CTQ scores, and subscale scores were assessed. Multiple comparisons were performed using a False discovery rate (FDR) < 0.05.

To enhance the robustness of our research findings, we conducted a sensitivity analysis. This involved reanalyzing the sample after excluding individuals with a family history of mental disorders.

## Result

### Demographic and clinical characteristics

Comprehensive demographic and clinical information are provided in **Table 1**. No statistically notable variations were observed in age, gender, or educational level across the groups. All intergroup comparisons were performed using Bonferroni correction for multiple comparisons. No statistically significant disparity in illness duration was observed between the MDD-CT and MDD-nCT groups.

**Table 1:** Demographic and clinical characteristics among four groups.

Characteristic		MDD		HC	F/t/χ2	p
	CT(n=48)	nCT(n=23)	CT(n=23)	nCT(n=74)		
Sociodemographic						
Gender (male/female)	8/40	4/19	10/13	23/51	7.532 ^a^	0.057
Age(years)	25.33 ± 4.95	27.22 ± 8.47	26.04 ± 6.89	27.46 ± 7.67	0.999 ^a^	0.395
Education (years)	15.25 ± 1.73	15.13 ± 2.85	16.30 ± 2.20	16.16 ± 2.54	2.45^a^	0.065
Duration (month)	6.32 ± 4.09	6.57 ± 3.75	–	–	0.00	0.620
Family history	4/44	1/22	–	–	0.377	0.539
HAMD-24	36.58 ± 8.36	34.65 ± 7.55	2.57 ± 2.37	2.06 ± 1.84	521.56	<0.001
HAMA	24.10 ± 6.92	21.48 ± 7.03	2.09 ± 2.15	1.50 ± 1.65	290.29	<0.001
CTQ						
Total	55.85 ± 13.68	36.74 ± 6.63	43.35 ± 8.24	29.65 ± 4.00	90.14	<0.001
Emotional abuse	11.83 ± 4.68	7.91 ± 2.21	8.04 ± 2.77	5.84 ± 1.32	39.98	<0.001
Physical abuse	7.75 ± 3.15	5.48 ± 0.73	6.70 ± 2.93	5.31 ± 0.60	15.13	<0.001
Sexual abuse	6.54 ± 3.49	5.22 ± 0.74	6.04 ± 2.90	5.16 ± 0.57	4.37	0.005
Emotional neglect	16.54 ± 3.70	10.04 ± 2.60	12.52 ± 4.80	7.58 ± 2.44	76.02	<0.001
Physical neglect	11.94 ± 3.59	7.04 ± 1.80	10.04 ± 2.38	5.76 ± 1.06	75.41	<0.001

Data are mean ± standard deviation for age, education year, HAMD-24, HAMA, disease duration, and CTQ scores. F/t/χ2: Variables of age, years of education, HAMD-24, HAMA, and CTQ assessments were tested by one-way ANOVA or Welch-ANOVA as indicated by F; ^a^analysis by one-way ANOVA; gender and family history were tested by chi-square test as indicated by χ^2^; disease duration was tested by two-sample t-test as indicated by t. Significant post-hoc tests (p < 0.05, Bonferroni corrected or Games-Howell corrected): HAMD-24: MDD-CT = MDD-nCT > HC-CT = HC-nCT; HAMA: MDD-CT = MDD-nCT > HC-CT = HC-nCT; EA: MDD-CT > MDD-nCT = HC-CT > HC-nCT; PA: MDD-CT = HC-CT > MDD-nCT = HC-nCT; SA: MDD-CT = HC-CT > MDD-nCT = HC-nCT; EN: MDD-CT > HC-CT > MDD-nCT > HC-nCT; PN: MDD-CT > HC-CT > MDD-nCT = HC-nCT; Total: MDD-CT > HC-CT > MDD-nCT > HC-nCT.

MDD, major depressive disorder; HC, healthy control; CT, childhood trauma; HAMD-24, 24-items Hamilton Depression Scale; HAMA, Hamilton Anxiety Scale; CTQ-EA, Childhood Trauma Questionnaire-emotional abuse; CTQ, Childhood Trauma Questionnaire; EA, emotional abuse; P, physical abuse; SA, sexual abuse; EN, emotional neglect; PA, physical neglect.

### Diagnosis and CT effects on clinical variables

A notable main effect of CT on anxiety levels was noted, suggesting that participants with CT exhibited more severe anxiety symptoms compared to those with none or mild CT. Additionally, the diagnosis of MDD demonstrated an important main effect on both depression and anxiety levels, with individuals in the MDD group exhibiting significantly higher levels of both compared to the healthy control group. No significant effects of CT on depression levels or CT × diagnosis interaction were observed (refer to **Table 2**).

**Table 2:** Diagnosis and childhood trauma effect on depression and anxiety.

Characteristic	MDD		HC		Effect of diagnosis		Effect of CT		Effect of diagnosis×CT	
	CT	nCT	CT	nCT						
	(n=48)	(n=23)	(n=23)	(n=74)	F(P)	Eta^2^	F(P)	Eta^2^	F(P)	Eta^2^
HAMD-24	36.58 ± 8.36	34.65 ± 7.55	2.57 ± 2.37	2.06 ± 1.84	1222.14 (<0.001)	0.88	1.63 (0.204)	0.01	0.57 (0.45)	0.00
HAMA	24.10 ± 6.92	21.48 ± 7.03	2.09 ± 2.15	1.50 ± 1.65	656.60 (<0.001)	0.80	3.933 (0.049)	0.02	1.484 (0.225)	0.00

Data are mean ± standard deviation for HAMD-24, and HAMA.

MDD, major depressive disorder; HC, healthy control; CT, childhood trauma; HAMD-24, 24-items Hamilton Depression Scale; HAMA, Hamilton Anxiety Scale.

### Diagnosis and CT effects on FA

**Figure 1:**
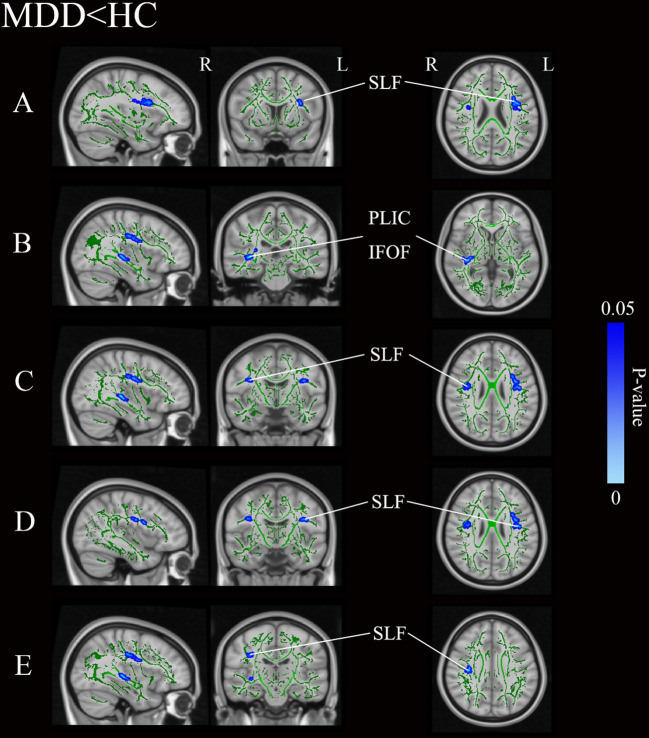
Group differences of TBSS analysis. Blue regions represent tracts with decreased FA in the MDD group compared with the HC group. **(A-E)** Represent five distinct cluster regions where significant differences between MDD and HCs were observed. MDD, major depressive disorders; HC, Healthy Controls; TBSS, tract-based spatial statistics; FA, fractional anisotropy; L, left hemisphere; R, right hemisphere; SLF, superior longitudinal fasciculus; PLIC, posterior limb of the internal capsule; IFOF, inferior fronto-occipital fasciculus.

**Figure 1** and **Table 3** illustrates regions where notable disparities in FA values were observed between the MDD and HCs. The brain areas showing decreased FA values in the MDD group included the right posterior limb of the internal capsule (PLIC), inferior fronto-occipital fasciculus (IFOF), and bilateral superior longitudinal fasciculus (SLF), totaling five clusters.

**Table 3:** Areas of reduced white matter FA in MDD patients compared with HC (p < 0.05, TFCE-corrected).

Anatomic location MDD vs HC	X	Y	Z	P-value	Voxel size
Superior longitudinal fasciculus L	-32	8	23	0.021	184
posterior limb of the internal capsule R/Inferior fronto-occipital fasciculus R	36	-27	2	0.017	179
Superior longitudinal fasciculus R	37	-7	26	0.034	88
Superior longitudinal fasciculus L	-39	-9	26	0.031	76
Superior longitudinal fasciculus R	35	-19	33	0.031	73

ANCOVA including covariates of age, sex, education year.

MDD, major depressive disorder; HC, healthy control; FA, fractional anisotropy; TFCE, threshold-free cluster enhancement; L, left hemisphere; R, right hemisphere.

As demonstrated in **Table 4**, a notable main effect of diagnosis on WM regions FA was noted. No significant effects of CT on WM regions FA or CT × diagnosis interaction were observed.

**Table 4:** Diagnosis and childhood trauma effect on white matter FA. (p < 0.05, TFCE-corrected).

Anatomic location	X	Y	Z	Effect of diagnosis	Effect of CT	Effect of diagnosis×CT
				P-value	P-value	P-value
Superior longitudinal fasciculus L	-32	8	23	0.021	1.000	1.000
posterior limb of the internal capsule R/Inferior fronto-occipital fasciculus R	36	-27	2	0.017	1.000	1.000
Superior longitudinal fasciculus R	37	-7	26	0.034	1.000	1.000
Superior longitudinal fasciculus L	-39	-9	26	0.031	0.998	1.000
Superior longitudinal fasciculus R	35	-19	33	0.031	1.000	1.000

ANCOVA including covariates of age, sex, education year. The statistical threshold was set at p<0.05, fully corrected for multiple comparison using TFCE across all white­ matter tracts in the whole­ brain analysis.

CT, childhood trauma; FA, fractional anisotropy; TFCE, threshold-free cluster enhancement; L, left hemisphere; R, right hemisphere.

**Figure 2:**
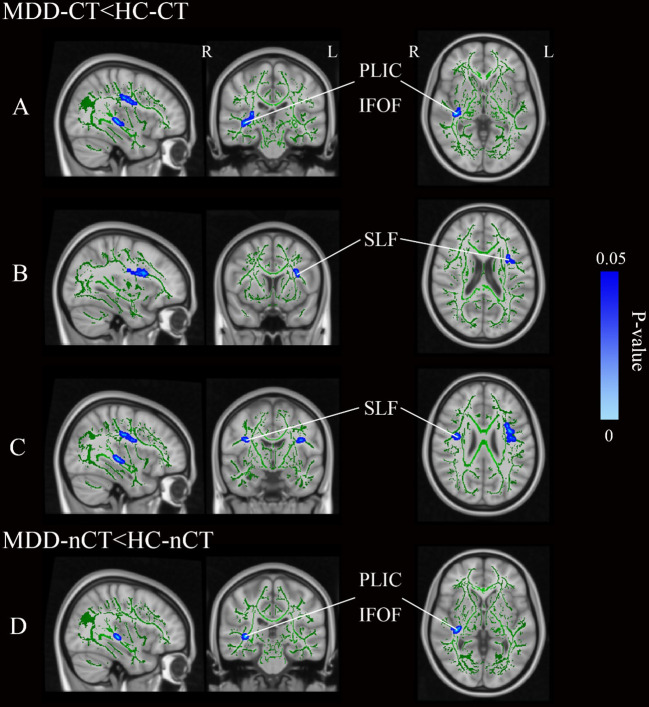
**(A–C)** Represent three distinct cluster regions where significant differences between MDD-CT and HC-CT were observed following simple effects analysis. **(D)** Represents a distinct cluster region where significant differences between MDD-nCT and HC-nCT were observed following simple effects analysis. Blue regions represent tracts with decreased FA (MDD-CT vs HC-CT and MDD-nCT vs HC-nCT). MDD, major depressive disorders; HC, Healthy Controls; CT, childhood trauma; FA, fractional anisotropy; L, left hemisphere; R, right hemisphere; SLF, superior longitudinal fasciculus; PLIC, posterior limb of the internal capsule; IFOF, inferior fronto-occipital fasciculus.

After simple effects analysis, as demonstrated in **Figure 2** and **Table 5**, the MDD-CT group showed notably lower FA values in the right PLIC (p<0.001), IFOF (p<0.001), and bilateral SLF (p<0.001) compared to the HC-CT group. Compared to the HC-nCT group, the MDD-nCT group showed lower FA values in the right PLIC and IFOF (p<0.001). No WM regions with increased FA values were found in the MDD group compared to HCs. No significant brain region differences were found between the MDD-CT group and the MDD-nCT group.

**Table 5:** The effect of diagnosis in CT and nCT groups separately.

Anatomic location	X	Y	Z	Pvalue	Voxel size
MDD-CT<HC-CT					
posterior limb of the internal capsule R/Inferior fronto-occipital fasciculus R	36	-25	-1	<0.001	171
Superior longitudinal fasciculus L	-32	9	20	<0.001	169
Superior longitudinal fasciculus R	35	-7	25	<0.001	86
Superior longitudinal fasciculus R	36	-18	32	<0.001	60
Superior longitudinal fasciculus L	-39	-9	26	<0.001	59
MDD-nCT<HC-nCT					
posterior limb of the internal capsule R/Inferior fronto-occipital fasciculus R	36	-27	1	0.005	59
Superior longitudinal fasciculus L	-40	-13	28	0.779	6
Superior longitudinal fasciculus R	37	-7	26	0.335	65
Superior longitudinal fasciculus R	35	-19	33	0.098	64
Superior longitudinal fasciculus L	-39	-9	26	0.325	59

A simple effects analysis including covariates of age, sex, education year (MDD-CT vs HC-CT and MDD-nCT vs HC-nCT).

MDD, major depressive disorder; HC, healthy control; CT, childhood trauma.

### Correlation analysis of WM with CT

**Figure 3:**
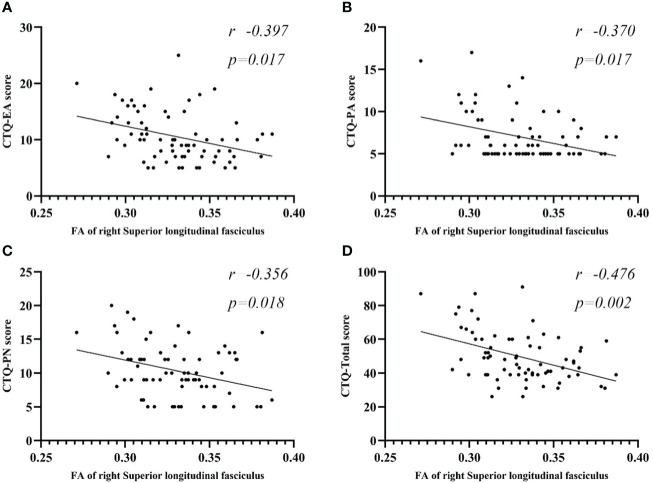
Scatter plots of partial correlation between FA regions and the target scales in MDD group. **(A)** Negative partial correlation between FA of right SLF and CTQ-EA score. **(B)** Negative partial correlation between FA of right SLF and CTQ-PA score. **(C)** Negative partial correlation between FA of right SLF and CTQ-PN score. **(D)** Negative partial correlation between FA of right SLF and CTQ-Total score. All correlations showed in this figure were constructed after controlling age, gender, years of education, and disease duration. MDD, major depressive disorder; FA, fractional anisotropy; CTQ, Childhood Trauma Questionnaire; CTQ-EA, emotional abuse subscale of childhood trauma questionnaire; CTQ-PA, physical abuse subscale of childhood trauma questionnaire; CTQ-PN, physical neglect subscale of childhood trauma questionnaire; CTQ-Total, Childhood Trauma Questionnaire total score; SLF, superior longitudinal fasciculus.

Partial correlation analysis between FA values and CTQ subscale scores, as well as total scores, are presented in **Figure 3** and **Table 6**. In the MDD group, Right SLF showed a negative correlation with EA (r=-0.397, p=0.017), PA (r=-0.370, p=0.017), PN (r=-0.356, p=0.018), and total score (r=-0.476, p=0.002). No significant correlations were found between the right PLIC, right IFOF, left SLF and CTQ subscale or total scores in the MDD group. There were also no notable correlations between the average FA values of each cluster and scores on the HAMD-24 and HAMA scales in the MDD group.

**Table 6:** Significant correlations between FA regions and CT subscales and total score in MDD.

	Regions of FA	r	p
MDD			
Emotional abuse	Superior longitudinal fasciculus R	-0.397	0.017
Physical abuse	Superior longitudinal fasciculus R	-0.370	0.017
Physical neglect	Superior longitudinal fasciculus R	-0.356	0.018
Total score of CTQ	Superior longitudinal fasciculus R	-0.476	0.002

Partial correlations between CT category with FA regions after controlling for age, gender, years of education and disease duration. Multiple comparisons were performed using a False discovery rate (FDR) < 0.05.

FA, fractional anisotropy; CT, childhood trauma; MDD, major depressive disorders; CTQ, Childhood Trauma Questionnaire; L, left hemisphere; R, right hemisphere.

### Sensitivity analysis

We excluded five individuals with a family history of mental disorders and employed the same analytical methods. The results of the sensitivity analysis remained robustly consistent with the original analysis findings (refer to **Tables 7**, **8**).

**Table 7:** Sensitivity analysis: Diagnosis and childhood trauma effect on white matter FA. (p < 0.05, TFCE-corrected).

Anatomic location	X	Y	Z	Effect of diagnosis	Effect of CT	Effect of diagnosis×CT
				P-value	P-value	P-value
Superior longitudinal fasciculus L	-32	8	23	0.030	1.000	1.000
posterior limb of the internal capsule R/Inferior fronto-occipital fasciculus R	36	-27	2	0.031	0.999	1.000
Superior longitudinal fasciculus R	35	-19	33	0.047	1.000	1.000

ANCOVA including covariates of age, sex, education year. The statistical threshold was set at p<0.05, fully corrected for multiple comparison using TFCE across all white­ matter tracts in the whole­ brain analysis.

CT, childhood trauma; FA, fractional anisotropy; TFCE, threshold-free cluster enhancement; L, left hemisphere; R, right hemisphere.

**Table 8:** Sensitivity analysis: areas of reduced white matter FA in MDD patients compared with HC (p < 0.05, TFCE-corrected).

Anatomic location MDD vs HC	X	Y	Z	P-value	Voxel size
Superior longitudinal fasciculus L	-32	8	23	0.030	100
posterior limb of the internal capsule R/Inferior fronto-occipital fasciculus R	36	-27	2	0.031	65
Superior longitudinal fasciculus R	35	-19	33	0.047	12

ANCOVA including covariates of age, sex, education year.

MDD, major depressive disorder; HC, healthy control; FA, fractional anisotropy; TFCE, threshold-free cluster enhancement; L, left hemisphere; R, right hemisphere.

## Discussion

This study aimed to identify neuroimaging evidence of structural brain changes in MDD patients who have experienced CT. We examined the effect of CT on MDD and the associations between CT, brain structural changes, and MDD. Our findings indicate a considerable main effect of diagnosis, with the MDD group exhibiting notable structural differences in the right IFOF, PLIC, and bilateral SLF compared to the HC group. CT did not show a significant main effect. Simple effects analysis revealed notable differences between the MDD-CT and HC-CT groups in the right IFOF, PLIC, and bilateral SLF, while the MDD-nCT and HC-nCT groups differed only in the right IFOF and PLIC. However, there were no interactive effects of CT × diagnosis. Moreover, a pronounced main effect of CT on anxiety symptoms was observed, with the CT group displaying heightened anxiety symptoms relative to the nCT group. Lastly, in the patient group, the right SLF was negatively correlated with EA, PA, PN, and total scores. This study is the first to comprehensively explore WM structural changes in drug-naive first-episode MDD patients who have experienced CT using the TBSS method.

Initially, our study found a reduction in FA in the right IFOF and PLIC in the MDD group compared to HCs. Our findings are supported by several MRI studies (30–32). Reduced FA is considered indicative of decreased WM organization, reduced axonal density, and myelination (33). Changes in these regions might be involved in the pathophysiology of depression. However, these differential changes were unrelated to the presence of CT. There were no significant differences in brain regions between the CT and nCT groups. This might indicate that the reduction in FA in the right IFOF and PLIC might be a specific change in the WM regions of MDD patients, independent of CT. These results differ from previous studies related to CT (15–17, 34, 35). However, Lim et al.’s study found that specific WM changes in the CT group were rectified in the subgroup analysis of unmedicated participants (34). Many studies have shown that medication can impact brain structure (30, 36). Therefore, the difference in our findings might be attributed to the inclusion of unmedicated participants. The IFOF, one of the longest association tracts in the human brain, links the occipital, frontal, and temporal lobes, and plays a role in emotional evaluation and visual perception regulation (37). The internal capsule, comprising multiple fiber tracts, connects the posterior limb with the parietal, temporal, occipital, and sensorimotor brain regions (38), and is associated with brain reward and self-stimulation processes (39). Structural changes in the IFOF and PLIC might be involved in the neurobiological mechanisms of MDD. We did not find a relationship between the IFOF, PLIC, and CT, which warrants further exploration in future research.

Secondly, we noted a marked decrease in FA in the bilateral SLF in the MDD group compared to HCs. Our study revealed structural changes in the SLF only between the MDD-CT and HC-CT groups, with no significant differences between MDD-nCT and HC-nCT groups. The SLF is an extensive fiber tract connecting almost all cortical areas of the lateral hemisphere, especially the frontal lobe with other cognitive or executive function brain regions. The SLF is associated with various cognitive and sensory functions, including language processing, attention control, working memory, executive function, visual and spatial processing (40, 41). Our findings in the SLF only validate prior research on MDD, suggesting that changes in SLF structure could be closely linked to the pathogenesis and progression of MDD. However, our study offers new insights regarding CT: the specific brain structural changes in MDD patients appear to be unrelated to CT, which differs from previous studies (15, 24, 42). We speculate that this might be due to related studies treating CT or MDD as isolated factors rather than examining them in conjunction. Additionally, clinical heterogeneity, including medication status, illness duration, and sample size, needs to be considered. These factors might collectively mediate the heterogeneity observed in our results. It is worth noting that CT and MDD are not entirely independent factors; CT, as a significant risk factor, has been proven to be involved in the development of MDD (43). Secondly, CT is an influencing factor in the pathogenesis of MDD but does not play a decisive role, which might partially explain the results we obtained after comparing the four groups. Finally, the significant differences between brain regions in MDD-CT and MDD-nCT might be due to differing levels of resilience among individuals with CT. Evidence suggests that CT can have varying impacts on an individual’s resilience (44), so those with experiences of CT might develop different outcomes (depression or non-depression) based on their resilience.

Although we did not identify significant brain structural changes related to CT experiences, our correlational analysis revealed a significant association between CT and FA values, as well as clinical symptoms. Our study found a notable main effect of CT on levels of anxiety. This suggests that CT might affect anxiety symptoms in both depressed and non-depressed individuals, with this impact being persistent and latent. Early life traumatic experiences might lead to higher levels of anxiety in adulthood. Our findings are supported by previous research indicating that CT experiences increase symptoms of depression, anxiety, and negative life event appraisal in adults (45). Furthermore, our exploration of the correlation between CT and WM fiber bundle FA values revealed negative correlations in the SLF with all trauma types except for the SA and EN. This suggests that CT may have a latent impact on the progression of MDD in patients. Adverse experiences in childhood, processed through individual sensory perceptions such as vision and hearing, could potentially affect the SLF, as it is involved in the processing of visual and auditory information. Consequently, we speculate that brain regions responsible for information processing may be susceptible to the effects of CT. This is supported by related research, which has found that CT predominantly affects several brain regions involved in cognitive and executive functions and information processing, such as areas for visual and auditory perception and language comprehension (46). Our findings of the correlation between CTQ scores and FA values in the SLF may indicate that CT subtly influences the developmental process of the SLF in the brains of MDD patients, thereby impairing cognitive and emotional functions and leading to more severe clinical symptoms.

Previous research has suggested that specific trauma types might have more severe impacts on individual brain structures: emotional neglect (35, 47), physical neglect (16, 35, 47), emotional abuse (8, 24), physical abuse (8, 13, 24), and sexual abuse (8, 13, 24). Our findings that EA, PA, PN and total scores predict a decrease in regional FA in the SLF suggests that the right SLF is susceptible to early adverse experiences. Future studies may concentrate on the association between different trauma types and the SLF. Moreover, the left SLF is not significantly correlated with CT scores. These differential results in the bilateral SLF suggest that we need to consider the effects of laterality. A recent study focusing on the SLF proposed that the SLF might play different roles in different hemispheres: the left SLF has a greater involvement in processing language, while the right SLF is closely related to visual spatial processing (41). A previous study supporting this view suggested that the right SLF facilitates rapid, prioritized visual-spatial processing in the right hemisphere (48). The greater correlation of the right SLF with CT might indicate a more substantial impact of CT experiences on individual visual processing-related brain regions. Additionally, in most individuals, the left hemisphere is notably less dominant in processing negative emotions (49). Therefore, CT might have a more widespread impact on the right hemisphere, known for its dominance in emotional processing, by affecting emotion regulation-related brain regions. Integrating these results, we speculate that CT experiences might subtly influence the right SLF in MDD patients, potentially altering functional networks. This alteration could impact the emotional and cognitive regulation functions of patients, potentially forming the basis for psychiatric symptoms. While these conclusions need further confirmation in future research, our findings might provide new insights into how and to what extent CT may influence the pathogenesis and progression of MDD.

Our study included drug-naive first-episode MDD patients and healthy individuals, and divided the patient and control groups into different subgroups based on CT experiences to explore the separate and interactive effects of CT and MDD diagnosis on individual brain WM fiber bundles. Our research provides new insights into the impact of CT experiences on MDD patients. However, this study has some limitations. Firstly, participants who experienced SA were quite rare in our study, which might limit our ability to detect brain changes related to this subscale. The reason for this phenomenon might be that many participants are unwilling to disclose. Future research should identify SA experiences or specifically study the impact of SA experiences on individual brain WM structure. Additionally, as the MDD-nCT patient group and HC-CT group had relatively small sample sizes, these results should be analyzed and interpreted cautiously, and future studies should include more samples. Then, our study focused solely on the FA. Although FA is widely used due to its sensitivity to microstructural changes (50), other DTI metrics such as mean diffusivity, radial diffusivity, and axial diffusivity also provide unique and complementary information about brain tissue characteristics. Future research could benefit from an integrated analysis incorporating these DTI metrics, which could lead to more comprehensive results. Lastly, the CTQ is a retrospective questionnaire, which may be subject to recall bias. Although previous research has shown that self-recollection can provide reliable information (51, 52), in MDD patients, due to their more negative self-evaluation and cognition, recollections of childhood experiences might be more negative. Future research can avoid this flaw through prospective studies or structured interviews.

## Conclusion

This study explored the specific changes in brain WM structure in drug-naive first-episode MDD patients who have experienced CT. MDD individuals exhibited reduced FA in the right IFOF, PLIC, and bilateral SLF compared to HCs. Notably, the right SLF showed a significant reduction in FA and negative correlation with CT. Our findings suggest that the brain development of MDD patients might be latently influenced by CT, potentially linked to individual differences in resilience, with the SLF playing a pivotal role. These findings help us understand the role of the SLF in the pathophysiology of MDD and the neurobiological mechanisms by which CT influences MDD.

## Data availability statement

The raw data supporting the conclusions of this article will be made available by the authors, without undue reservation.

## Ethics statement

The studies involving human participants were reviewed and approved by the Ethics Committee of the Renmin Hospital of Wuhan University (WDRY2020-K236). The studies were conducted in accordance with the local legislation and institutional requirements. The participants provided their written informed consent to participate in this study. Written informed consent was obtained from the individual(s) for the publication of any potentially identifiable images or data included in this article.

## Author contributions

HZ: Conceptualization, Data curation, Formal analysis, Investigation, Methodology, Validation, Visualization, Writing – original draft, Writing – review & editing. BR: Conceptualization, Data curation, Formal analysis, Investigation, Methodology, Validation, Writing – review & editing. GG: Conceptualization, Data curation, Investigation, Methodology, Validation, Writing – review & editing. MZ: Data curation, Investigation, Methodology, Validation, Writing – review & editing. JH: Data curation, Investigation, Validation, Writing – review & editing. NT: Data curation, Investigation, Resources, Writing – review & editing. LB: Data curation, Investigation, Resources, Writing – review & editing. LX: Conceptualization, Methodology, Resources, Supervision, Validation, Writing – review & editing. GW: Conceptualization, Funding acquisition, Project administration, Resources, Supervision, Validation, Writing – review & editing.
